# Risk of Motor Vehicle Collisions and Culpability among Older Drivers Using Cannabis: A Meta-Analysis

**DOI:** 10.3390/brainsci13030421

**Published:** 2023-02-28

**Authors:** Arun Chinna-Meyyappan, Hui Jue Wang, Kritleen K. Bawa, Edward Ellazar, Emilie Norris-Roozmon, Gary Naglie, Nathan Herrmann, Judith L. Charlton, Sjaan Koppel, Saulo Castel, Krista L. Lanctôt, Mark J. Rapoport

**Affiliations:** 1 Neuropsychopharmacology Group, Sunnybrook Research Institute, 2075 Bayview Avenue, Toronto, ON M4N 3M5, Canada; 2 Department of Pharmacology and Toxicology, University of Toronto, 27 King’s College Circle, Toronto, ON M5S 1A1, Canada; 3 Hurvitz Brain Sciences Program, Sunnybrook Research Institute, 2075 Bayview Avenue, Toronto, ON M4N 3M5, Canada; 4 Canadian Cancer Research Trials Group, Queen’s University, 99 University Avenue, Kingston, ON K7L 3N6, Canada; 5 Department of Medicine and Rotman Research Institute, Baycrest Health Sciences, 3560 Bathurst Street, Toronto, ON M6A 2E1, Canada; 6 Department of Medicine and Institute of Health Policy, Management and Evaluation, University of Toronto, 27 King’s College Circle, Toronto, ON M5S 1A1, Canada; 7 Department of Psychiatry, University of Toronto, 27 King’s College Circle, Toronto, ON M5S 1A1, Canada; 8 Department of Psychiatry, Sunnybrook Health Sciences Centre, 2075 Bayview Avenue, Toronto, ON M4N 3M5, Canada; 9 Monash University Accident Research Centre, Monash University, Wellington Road, Clayton, VIC 3800, Australia

**Keywords:** cannabis, older adults, driving, motor vehicle collision, culpability, THC, road traffic crashes, delta-9-tetrahydrocannabinol

## Abstract

Limited studies have investigated the effects of cannabis use on driving among older adults, who represent the fastest growing segment of drivers globally. We conducted a systematic review and meta-analysis to evaluate the effects of delta-9-tetrahydrocannabinol (THC) exposure on risks of (1) motor vehicle collisions (MVC) and (2) culpability for MVCs among adults 50 years and older. Three reviewers screened 7022 studies identified through MEDLINE, EMBASE, CENTRAL, and PsycINFO. Odds Ratios (OR) were calculated using the Mantel-Haenszel method in Review Manager 5.4.1. Heterogeneity was assessed using I^2^. The National Heart, Lung, and Blood Institute tool was used to assess the quality of each study. Seven cross-sectional studies were included. Three studies evaluated culpability while four evaluated MVC. The pooled risk of MVC was not significantly different between THC-positive and THC-negative older drivers (OR, 95% CI 1.15 [0.40, 3.31]; I^2^ = 72%). In culpability studies, THC exposure was not significantly associated with an increased risk of being culpable for MVC among adults over the age of 50 (OR, 95% CI 1.24 [0.95, 1.61]; I^2^ = 0%). Inspection of funnel plots did not indicate publication bias. Our review found that THC exposure was not associated with MVC involvement nor with culpability for MVCs.

## 1. Introduction

Older adults are projected to become the fastest-growing segment of drivers as a consequence of population aging across the globe [1,2]. In 2020, there were 48 million licensed drivers over the age of 65 in the United States alone, representing a 68% increase from 2000 [3]. The aging process can be accompanied by a variety of physical and psychological conditions that can lead to deterioration in the motor, perceptual and cognitive skills required to operate a vehicle [2]. Visual impairment [4], musculoskeletal conditions [5], and certain cardiovascular diseases [6] are known to be highly prevalent in older populations and can negatively impact driving ability [7]. Moreover, higher fatality rates following motor vehicle collisions (MVC) have also been reported for older drivers over the age of 70, possibly due to their increased vulnerability to injury [8,9]. While poorer driving outcomes may be partly attributed to the comorbidities associated with aging [10], modifiable factors such as substance use have also been known to further exacerbate these risks [11].

Cannabis is one of the most frequently detected drugs in drivers involved in MVCs, second only to alcohol [12,13]. The psychoactive component of cannabis, delta-9-tetrahydrocannabinol (THC), has been shown to decrease reaction times, motor coordination, and sensory perception [14], all of which could impair an individual’s driving ability. With cannabis legalization becoming increasingly commonplace globally [15,16], there have been concerns that ready access to this previously illicit drug could lead to more impaired drivers on the road and subsequently more MVC-related injuries and fatalities [16,17,18]. For instance, one study found that the rate of moderately injured drivers with a THC level of at least 2 ng/mL more than doubled following cannabis legalization in Canada in 2018 [19]. Of particular relevance is that the subgroup of adults aged 50 and over had the highest increase in cannabis use prevalence in that study.

While not specific to older populations, some additional evidence from epidemiologic research suggests that cannabis use may adversely affect driving performance. In a meta-analysis that reviewed both observational and simulation studies of cannabis use and driving among all age groups, Li et al. concluded that cannabis use was associated with a significantly higher risk of crash involvement [12]. Similar results were described by another meta-analysis of observational studies by Asbridge et al. of drivers of all age groups, which reported that acute cannabis consumption nearly doubled the risk of being in an MVC involving serious injury or death [20].

Among collision-involved drivers, another important question to dissect is whether individuals who consume and drive under the influence of cannabis are more likely to be deemed responsible for their crashes. Researchers have attempted to address this issue through a technique called culpability analysis, or responsibility analysis [21]. In culpability studies, all drivers have been involved in MVCs and are subsequently required to undergo standard legal or medical investigations [22,23,24]. Such studies are well suited to examine transient exposures such as acute cannabis use, particularly in cases where participant refusal rates are high [25]. Historically synonymous with legal liability, the term culpability has evolved to characterize a broader range of study designs in modern road safety research [25]. For instance, some culpability studies take into account a variety of external factors surrounding an MVC and apply a validated scoring system to determine driver responsibility [26]. One study by Drummer et al. that used this paradigm found the odds of being culpable were 1.9-fold higher among THC-positive drivers of all ages in comparison to drug-free controls [26]. Conversely, Longo et al. found that the culpability rate of drivers of all ages who were THC-positive did not differ significantly from that of drug-free control drivers [22].

Given these mixed findings, the increasing use of cannabis in older adults, and the relative paucity of studies involving this age group, there is a clear need for further research to inform the development of age-specific guidelines and recommendations related to cannabis use and driving. To address this issue, the current systematic review and meta-analysis aimed to consolidate the existing evidence on the impact of THC exposure on two key driving outcomes, MVC risk, and culpability, in older adults.

## 2. Materials and Methods

### 2.1. Search Strategy

This review adhered to the Preferred Reporting Items for Systematic Reviews and Meta-Analyses (PRISMA) guidelines. Original research studies published in peer-reviewed journals that reported on the use of cannabinoids in older drivers against a comparison group were included. For the purposes of this study, older drivers were defined as adults aged 50 years and older, a cut-off that, while younger than the more conventional cut-off of 65 years and older, has been commonly used in studies of drug use in older adults not focused on MVCs [27,28]. Studies needed to either restrict their analyses to groups of adults ages 50 years and over, or to have subgroups in that age range. When studies included adults aged 50 years and over but data specific to that subgroup were not published in the manuscripts, we contacted the authors to obtain this information. Reviews, case studies, commentaries, and studies not available in English or in full-text form were excluded.

This meta-analysis was registered to the National Institute for Health Research registry for systematic reviews PROSPERO (ID: CRD42021252244, registered 30 April 2021) prior to commencing the search strategy. We conducted a systematic review of primary MVC research studies identified in epidemiological/observational, on-road, simulator, or culpability studies, for MVCs associated with cannabinoid consumption in older adult drivers. Searches were completed in January 2021 on the following databases: MEDLINE, CENTRAL, EMBASE, and PsycINFO. Search terms used are included in Appendix A-Search Strategies. Reference lists of included studies and recently published reviews were scanned for additional relevant citations.

### 2.2. Data Synthesis and Quality Assessment

Endnote 20 was used to remove duplicate studies after retrieval from databases. Studies then underwent title, abstract, and full-text screening by four reviewers using the Covidence tool. Three reviewers (EE, ENR, MS) independently screened studies using a priori inclusion and exclusion criteria and conflicts were resolved as a team. Two authors extracted data from the included studies (AC and HJW). One study did not report numerical data and presented results only in the graphical form [29]. This data was converted to usable data by one author (AC) using the WebPlotDigitizer software, which provided raw numerical values from the bar graphs based on the size of the bars in relation to the *y* axis. Three reviewers (AC, HJW, and MR) evaluated the risk of bias of included studies using the National Heart Lung and Blood Institute (NHLBI) Quality Assessment Tool for Observational Cohort and Cross-Sectional Studies [30]. NHLBI is one of the National Institutes of Health (NIH) in the United States of America [30].

### 2.3. Statistical Analysis

A high variability was expected between studies due to methodological differences; hence, random effects meta-analyses were used to assess the culpability and MVC risk between THC-positive and THC-negative older adult drivers. In particular, differences in the methods used to determine MVC and THC exposure and in the time frames between exposures and outcomes are some of the variables that could potentially contribute to variability and heterogeneity between studies. Odds ratios and 95% confidence intervals were calculated and reported for the two primary outcomes: MVC risk and culpability. The odds ratios define a ratio of the probability of an outcome occurring (culpability/MVC) to the probability of the outcome not occurring in the two groups (i.e., THC-positive and THC-negative older adult drivers).

Heterogeneity was measured using the Cochran Q test, and a *p* value of <0.05 indicated the presence of significant heterogeneity. Heterogeneity was quantified using I^2^, where an I^2^ > 50% indicated substantial heterogeneity in a meta-analysis. A limited number of studies in both meta-analyses restricted the use of pre-planned meta-regressions and sub-group analyses to explore possible sources of heterogeneity.

Sensitivity analyses were performed by removing studies with a high potential risk of biases where permitted. Funnel plots were used to qualitatively assess publication bias by visual inspection. All statistical analyses were performed using RevMan 5 statistical software.

## 3. Results

**Figure 1:**
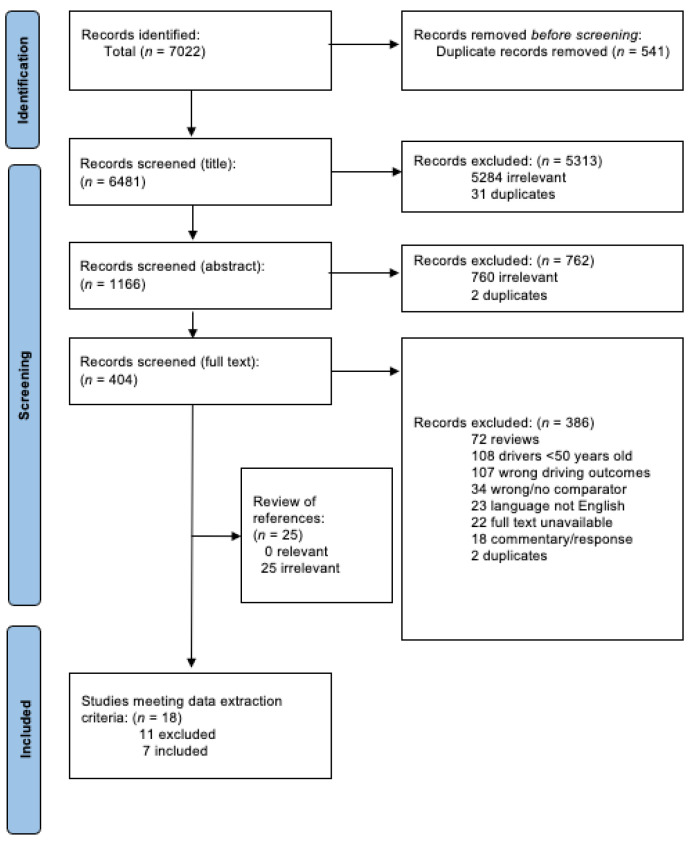
Flowchart of literature search and screening process using PRSIMA.

We identified 7022 studies from the four databases, resulting in 6481 studies after duplicates were removed (Figure 1). We excluded 5284 studies during title screening due to irrelevance and 762 studies met exclusion criteria. Full text screening was completed for 404 studies, and 18 studies initially appeared to meet the criteria for data extraction.

During the data extraction process, 11 additional studies were excluded due to MVC or culpability data being unavailable for older adults. Data extraction was completed for seven studies [29,31,32,33,34,35,36], all of which were observational in design. Reference list scanning did not result in the inclusion of additional studies. Data request letters were sent to corresponding authors of studies that did not report findings specific to older adults. Four of 47 authors replied with the relevant data: Jørgenrud et al. [32], Brubacher et al. [34], Poulsen et al. [35], and Chihuri and Li [36] all provided usable data specific to adults aged 50 and older.

### 3.1. Overview of Included Studies

Studies included in this meta-analysis were published between 2010 and 2021 and ranged in sample size from 1000 to 11,712 individuals. After thoroughly reviewing the methodology of the included studies, we found that all of them used study designs whereby exposure and outcome data were measured at approximately the same point in time. As such, we concluded that it would be reasonable to classify all seven as being cross-sectional, which sometimes differed from the original authors’ categorization. Specifically, Brubacher et al. [34] considered their study as having a prospective case-control design, while Kalantari Meibodi et al. [29] described their study as being both a cross-sectional and case-control study. Details on the quality assessment of included studies can be found in Table 1. 

Were key potential confounding variables measured and adjusted statistically for their impact on the relationship between exposure(s) Overall, five studies used objective measures of THC exposure (e.g., blood, oral fluid, or urine samples) [31,32,34,35,36], one study used a subjective measure (e.g., self-reported THC use) [33], and one study used a combination of both [29]. Four of the seven studies assessed MVC risk as the primary outcome [29,31,32,33], with two studies using subjective measures of MVC (e.g., self-reported crash involvement) [32,33], and two studies using objective measures of MVC (e.g., MVC data collected at the scene of collision) [29,31]. The remaining three studies evaluated culpability using both subjective and objective data obtained from police crash reports and other supporting documents [34,35,36]. Two studies determined driver responsibility by applying validated scoring systems and assessing the extent to which external factors contributed to each crash [34,35]. Within that framework, lower scores denoted a greater degree of personal responsibility and, in turn, culpability. The third culpability study counted driving errors as a proxy measure for culpability [36].

### 3.2. Studies of THC Exposure and MVC Risk

The study by Johnson et al. [31] aimed to investigate whether the potential effects of cannabis use on MVC risk differed between younger and older drivers. Older drivers were defined as adults aged 50 and over. This was the only included study in which MVC data were collected directly at the scene of the collision. Drivers who were medically fit to be interviewed were asked to provide oral fluid and blood samples; non-crash control drivers were sampled one week later at the same locations and times of day. Upon further analysis of the fitted models with significant interaction effects between age and THC exposure, the authors found that the MVC risk associated with THC increased with age. There was a significant association at age 64 (F (1, 6757) = 3.8, *p* = 0.049), which was even greater at age 76 (F (1, 6757) = 4.8, *p* = 0.028) (Table 2). Accordingly, they concluded that THC exposure was associated with an increased risk of crash involvement in older adults.

Kalantari Meibodi et al. [29] conducted a study to investigate drug use in non-fatally injured drivers of motor vehicles. Authors compared drug use in 500 drivers hospitalized following a motor vehicle crash to 500 drivers hospitalized in the same emergency room for non-traumatic reasons. A two-step process was used to determine exposure to THC and other illicit substances. First, patients were asked whether they had used any drugs during the 72 h prior to their hospital referral. For patients who claimed to have a negative history, urine samples were then collected and analyzed for illicit drugs. Using the data in the manuscript specific to adults over the age of 55, we showed that THC exposure was associated with lower odds of being involved in an MVC in this age group (OR, 95% CI 0.32 (0.11, 0.92)) (Table 2).

The study by Jørgenrud et al. [32] investigated the relationships between alcohol use, drug use, speeding, and MVC risk. Data collection was completed in collaboration with the police who stopped drivers at random for roadside assessments. Participants were asked to provide oral fluid samples and complete a survey regarding crash involvement in the past two years. After obtaining additional data from the authors following a data request, we determined that THC exposure was not significantly associated with odds of MVC involvement (OR, 95% CI 2.28 (0.12, 44.47)) for adults aged 50 and older (Table 2).

In their study, Mann et al. [33] stratified participants by age and allowed separate analyses to be conducted for drivers aged 18–34 years, 35–54 years, and 55 years and older. In this study, a telephone survey was conducted to gather data on involvement in both MVCs and drug use in the last 12 months. Within the older adults (55+ years) subgroup, none of the predictors, including cannabis use, were associated with collision risk (OR, 95% CI 1.18 (0.32, 4.36)) (Table 2).

### 3.3. Studies of THC Exposure and Driver Culpability

The three studies on cannabis use and culpability in MVC reported results for all age groups and did not perform separate analyses for subgroups of older adults [34,35,36]. Data for older adults, specifically, were obtained from each study’s corresponding author via data requests.

Brubacher et al. [34] investigated whether THC-positive drivers injured in MVCs were more likely to be responsible for their crashes than sober drivers among all age groups. They prospectively sampled drivers from trauma centers and analyzed blood samples for medications and illicit drugs. Police reports were assessed using a validated scoring system to determine culpability. Based on the data provided by the authors for adults aged 50 and older, we found that THC exposure did not have a significant effect on driver culpability in this age group (OR, 95% CI 1.21 (0.49, 2.96)) (Table 3).

The second study by Poulsen et al. [35] aimed to determine the influences of cannabis, alcohol, and other psychoactive drugs on the culpability of drivers killed in MVCs. The authors used a validated scoring methodology similar to that of Brubacher et al. [34] to assign culpability and assayed blood samples for the presence of various drugs. After analyzing the data from the authors for adults aged 50 and older, we determined that THC exposure was not significantly associated with culpability among older adults (OR, 95% CI 1.12 (0.12, 10.30)) (Table 3).

Lastly, the study by Chihuri and Li [36] used a pair-matched design to explore the direct and indirect effects of cannabis use with and without alcohol on crash culpability in fatal 2-vehicle MVCs. Authors retrospectively acquired driver crash data and toxicological testing results from the Fatal Analysis Reporting System (FARS) for 5856 pairs of drivers of all ages. Analysis of author-provided data on the subgroup of adults aged 50 and above revealed that THC exposure did not significantly affect the risk of being deemed culpable for an MVC in this age group (OR, 95% CI 1.24 (0.94, 1.64)) (Table 3).

### 3.4. Other Features of Studies

i. Confounding Variables

Evidence from the literature suggests that age, sex, and exposure to alcohol and other drugs are key factors that may affect MVC risk and culpability [37,38,39,40,41]. In other words, they are potential confounding variables that should be accounted for in the analyses. Six of the seven included studies controlled for alcohol use or blood alcohol level [31,32,33,34,35,36]. However, only three of the included studies controlled for polydrug use in their analyses [32,34,35]. Overall, Johnson et al. [31] had the most comprehensive adjustments for potential confounders. They included assessments of driving exposure and were the only study to consider the potential effects of ethnicity and race. With driving exposure, for instance, they included an “annual miles” variable with three distinct levels and a “miles from home” variable with four levels. Mann et al. [33] also controlled for driving exposure by measuring kilometers driven in a typical week. Two studies adjusted for driving history (e.g., speeding, license suspension) [32,36]. Income and marital status were addressed by only one study [33]. Finally, all but one study [29] reported controlling for age and sex [31,32,33,34,35,36].

ii. Measures of THC Exposure

THC and its metabolites can be detected in the blood, saliva, and urine using various immunoassay and chromatographic techniques [42]. Four of the seven included studies used blood samples to detect the presence of THC [31,34,35,36]. Of these, Brubacher et al. [34] and Poulsen et al. [35] stated that their limits of detection were 0.2 ng/mL and 0.3 ng/mL, respectively. Two studies used oral fluid samples [31,32]. Only two of the seven studies quantified THC concentrations in the samples rather than simply reporting the absence or presence of THC [34,35]. One study used urine samples [29], and one study used self-report questionnaire data [33].

iii. Timeframe of THC Exposure

When cannabis is inhaled, the psychotropic effects of THC peak around 15 to 30 min after use and generally resolve by 4 h [43,44]. In contrast, orally administered THC induces its maximum psychotropic effects after 2 to 3 h, which then linger for up to 12 h [44]. For our meta-analysis, studies were deemed to have measured THC exposure within a reasonable time frame for detecting potential cannabis-induced impairment if they collected biological samples within 6 h of an MVC. Overall, only three of the seven included studies met this criterion [31,34,35]. Johnson et al. [31] collected blood samples on the scene of the crashes and had the most temporally relevant measurements of THC exposure. Both Brubacher et al. [34] and Poulsen et al. [35] specified collecting blood samples within 6 and 4 h of an MVC, respectively. Chihuri and Li [36] did not clearly state whether blood samples were taken within a certain number of hours following collisions, though this could be inferred for drivers who were promptly taken to a hospital. Kalantari Meibodi et al. [29] asked participants about drug use in the last 72 h. Jørgenrud et al. [32] collected oral fluid samples and used self-reports to determine MVC involvement in the past 12 months, which meant that there was no way to determine whether an MVC occurred soon after THC exposure. Mann et al. [33] had the least reliable method of measuring THC exposure as they used self-reports to assess drug use in the past 12 months.

### 3.5. Meta-Analysis of Risk of MVC with THC

**Figure 2:**
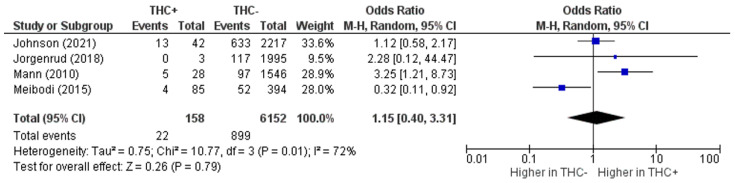
Pooled odds ratio (95% CI) of motor vehicle collision (MVC) risk associated with THC exposure in older drivers over the age of 50 [29,31,32,33].

The first of our two primary analyses examined the effect of THC exposure on MVC risk in older drivers and included four studies [29,31,32,33]. The pooled risk of MVC was not significantly different between older adult drivers who were exposed to THC and those who were not (OR, 95% CI 1.15 (0.40, 3.31)) (Figure 2). We noted significant heterogeneity among the studies (I^2^ = 72%, *p* = 0.01) (Figure 2). Three of the four studies point in the direction indicating that cannabis use is associated with an increased risk of MVCs, [31,32,33] though Mann et al. [33] was the only study that had statistically significant results. Conversely, Kalantari Meibodi et al. [29] concluded that MVC risk was significantly greater among THC-negative drivers compared to THC-positive drivers. Confidence intervals varied dramatically between studies with Kalantari Meibodi et al. [29] having a relatively small range and Jørgenrud et al. [32] having a very large range. Based on visual inspection of the funnel plot (Figure S1), there did not appear to be any publication bias among the studies of MVC risk.

**Figure 3:**
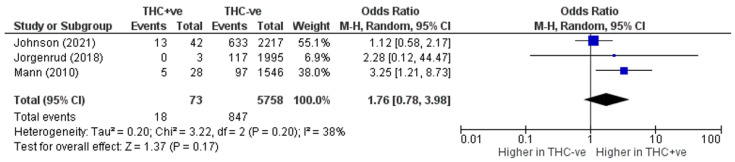
Sensitivity analysis with pooled odds ratio (95% CI) of motor vehicle collision (MVC) risk associated with THC exposure after removing Kalantari Meibodi et al. [31,32,33].

After performing a sensitivity analysis by excluding Kalantari Meibodi et al., the only study showing a significant inverse trend, heterogeneity was reduced to 38%. The summary cannabis-crash OR increased numerically, but remained statistically insignificant (OR, 95% CI 1.76 (0.78, 3.98)) (Figure 3).

### 3.6. Meta-Analysis of MVC Culpability with THC

**Figure 4:**
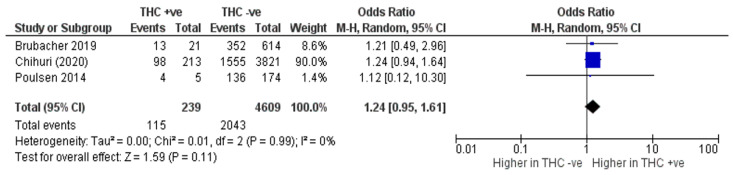
Pooled odds ratio (95% CI) of culpability risk associated with THC exposure in older drivers over the age of 50 [34,35,36].

For our second primary analysis which included three studies [34,35,36], we found that THC exposure was not significantly associated with an increased risk of being culpable for an MVC among adults over the age of 50 (OR, 95% CI 1.24 (0.95, 1.61)) (Figure 4). The MVC risk studies displayed no heterogeneity (I^2^ = 0%, *p* = 0.99) and all showed results in the expected direction (Figure 4). Similar to the MVC risk analysis, the widths of confidence intervals differed substantially. Studies on culpability did not display publication bias upon inspection of the funnel plot (Figure S2).

## 4. Discussion

This systematic review and meta-analysis identified seven studies that investigated MVC risk or culpability in older adult drivers aged 50 years and older who used cannabis. We found that THC exposure was not associated with MVC risk or culpability.

Overall, our findings were incongruent with previous literature that documents positive associations with respect to the effects of THC exposure on MVC risk in the general population (i.e., studies that did not focus on older adults). For instance, a widely cited systematic review and meta-analysis by Asbridge et al. reported that acute cannabis use was associated with a nearly two-fold higher MVC risk among all age groups [20]. Subgroup analyses revealed that this association was particularly salient in studies of fatal MVCs compared to studies involving non-fatal injuries. Supporting and extending those findings, a meta-analysis by Li et al., reported that cannabis use was associated with 2.66 times greater odds of being involved in an MVC [12]. In addition, those authors concluded that MVC risk increased in a dose-response manner with concentrations of THC-COOH, the main metabolite of THC, and with frequency of self-reported cannabis use. An important caveat of those findings, as noted by Rogeberg and Elvik [45], is that both reviews selected unadjusted odds ratios of MVC risk from individual studies even when adjusted odds ratios were available. By disregarding potential confounding factors, it is possible that estimates of cannabis related MVC risk were inflated. Indeed, meta-analyses that were careful to use adjusted odds ratios have reported more conservative estimates of MVC risk associated with cannabis use [45,46]. Indirectly, our review serves as a reminder that consensus has yet to be reached regarding the MVC risk associated with cannabis use.

Compared to MVC risk, there is a lack of high-level evidence on the relationship between cannabis use and driver culpability, and results from the limited available literature are conflicting. A recent systematic review and meta-analysis by White and Burns initially found that cannabis use was associated with a 1.37 times greater likelihood of being culpable for an MVC in studies that did not focus on older adults [47]. However, after adjusting for directional biases, they obtained a summary odds ratio of 0.68, which suggested that cannabis use has a protective effect. Given these seemingly contradictory findings, there was inadequate evidence to conclude that cannabis use has a significant effect on culpability in either direction. Thus, it is clear that the high variability among studies of cannabis use and driving outcomes is not a new phenomenon and that study biases can severely distort estimates of cannabis related MVC risk and culpability risk. With this in mind, the lack of significance for the results of this meta-analysis was not unexpected.

Although the studies included in this meta-analysis had fairly large sample sizes, none of them focused exclusively on older adults, but rather had subgroups of older adults. Many of those studies also tested for a wide range of different drugs or substances and not only cannabis. Consequently, the number of cases included in our analyses may have been too low to detect a significant effect as there were relatively few drivers who were both over the age of 50 years and exposed to THC. From our meta-analyses, there is evidence that statistical significance may have emerged if a larger number of studies were available and included in the analysis. Notably, there was 0% heterogeneity among culpability studies, which all showed results in the same direction.

The results of this systematic review and meta-analysis need to be considered in light of several limitations. All seven of the included studies were cross-sectional in nature. As such, it was not possible to draw a causal relationship between cannabis use and MVC risk or culpability. Furthermore, the studies included in this review assessed THC exposure through biological sampling or self-report questionnaires, neither of which are indicative of THC impairment at the time of operating a motor vehicle [48]. Plasma concentrations of THC generally vary around 1–35 ng/mL for drivers under the influence of cannabis [49] and from 1–100 ng/mL in drivers involved in fatal MVCs [26]. Despite those wide ranges, THC concentrations of as little as 7–10 ng/mL have been used as legal thresholds for cannabis impairment in several jurisdictions [50]. Our meta-analysis cannot comment on the validity of legislation based on legal thresholds for cannabis impairment. However, the two studies that quantified THC exposure by level in our review also used low thresholds for cannabis impairment, starting at 0.2 ng/mL [34,35]. A study assessing THC concentrations in chronic users of cannabis found that 50% of participants had concentrations greater than 1 ng/mL, ranging from 1.2–5.5 ng/mL, seven days after cessation of cannabis use [51]. Evidently, the levels of THC measured in these included studies cannot indicate active cannabis impairment or even recent use while driving.

Four of the included studies did not control for polydrug use in their analyses, one of which also did not control for alcohol. This limits our ability to associate driving outcomes such as MVC and culpability with cannabis impairment alone. The additive effects of cannabis and alcohol on driving impairment are well established in the literature, both in simulation studies [52] and on-road studies [22,24,53]. Studies have also found a potential synergistic effect between cannabis and other drugs, such as cocaine and MDMA [54]. When left uncontrolled, this effect introduces a bias that may overestimate the effect of cannabis on MVC risk.

There were also no studies that included subgroups specifically 65 years and over, a more conventional definition of older adults, and all of them used the cut-off of 50 or 55 years and over. This provides an important limitation to our understanding of the impact of cannabis on road safety in older adults.

## 5. Conclusions

The present systematic review and meta-analysis found that THC exposure was not associated with MVC involvement in older adults, nor with culpability when involved in an MVC. However, it would be an oversimplification to conclude that cannabis use does not pose a serious safety risk to older drivers. Rather, our results are likely a reflection of the many methodological limitations of studies included in our review. Future studies should strive to use objective measures to determine THC exposure and MVC involvement in order to reduce potential response bias. Additionally, cannabis-associated impairment can vary vastly among individuals based on a variety of factors. It would be more meaningful to quantify THC concentrations instead of measuring THC exposure as a binary outcome so that the dose-response effect of cannabis on driving outcomes can be determined. To address heterogeneity within the study population, researchers could stratify participants into subgroups based on characteristics known to affect THC pharmacokinetics such as age, frequency of cannabis use (e.g., occasional, chronic), and route of administration (e.g., inhalation, ingestion) [44,51]. In short, a greater quantity and quality of research is still needed, despite best efforts to date. Recent literature suggests that legalization and broader social acceptance of cannabis use have sparked increased use in older adults, which could facilitate cannabis-related research [19,55,56]. Thus, we are optimistic that future researchers will be in a more advantageous position to evaluate the impact of THC exposure on driving outcomes such as crash risk and culpability.

## Figures and Tables

**Table 1:** NHLBI Risk of Bias Table for Cross-sectional and cohort studies.

	Question Number														
Author (Year)	1	2	3 *	4	5	6	7	8	9	10 *	11	12	13 *	14	Quality Rating
*Culpability Studies*															
Brubacher	Yes	Yes	NA	Yes	Yes	No	No	Yes	Yes	NA	Yes	Yes	NA	Yes	“Good”
Chihuri	Yes	Yes	NA	Yes	No	No	No	No	Yes	NA	Yes	Yes	NA	Yes	“Fair”
Poulsen	No	Yes	NA	Yes	No	No	No	Yes	Yes	NA	Yes	Yes	NA	Yes	“Good”
*MVC Risk Studies*															
Johnson	Yes	Yes	NA	Yes	No	No	No	No	Yes	NA	Yes	No	NA	Yes	“Good”
Jørgenrud	Yes	Yes	NA	Yes	No	No	No	No	Yes	NA	No	No	NA	Yes	“Poor”
Mann	Yes	Yes	NA	Yes	No	No	No	No	No	NA	No	No	NA	Yes	“Poor”
Kalantari Meibodi	Yes	Yes	NA	Yes	No	No	No	No	No	NA	Yes	No	NA	No	“Poor”

NA = Not applicable. * Questions are not applicable to cross-sectional studies. Questions: (1) Was the research question or objective in this paper clearly stated? (2) Was the study population clearly specified and defined? (3) Was the participation rate of eligible persons at least 50%? (4) Were all the subjects selected or recruited from the same or similar populations (including the same time period)? Were inclusion and exclusion criteria for being in the study prespecified and applied uniformly to all participants? (5) Was a sample size justification, power description, or variance of effect estimates provided? (6) For the analyses in this paper, were the exposure(s) of interest measured prior to the outcome(s) being measured? (7) Was the timeframe sufficient so that one could reasonably expect to see an association between exposure and outcome if it existed? (8) For exposures that can vary in amount or level, did the study examine different levels of exposure as related to the outcome (e.g., categories of exposure, or exposure measured as a continuous variable)? (9) Were the exposure measures (independent variables) clearly defined, valid, reliable, and implemented consistently across all study participants? (10) Was the exposure(s) assessed more than once over time? (11) Were the outcome measures (dependent variables) clearly defined, valid, reliable, and implemented consistently across all study participants? (12) Were the outcome assessors blinded to the exposure status of the participants? (13) Was loss to follow-up after baseline 20% or less? (14) Were key potential confounding variables measured and adjusted statistically for their impact on the relationship between exposure(s)?

**Table 2:** Characteristics and Results of the Included MVC Risk Studies.

Author (Year), Quality Rating	Country (Dates)	Group Characteristics	THC Exposure Ascertainment	MVC Ascertainment	Key Findings in Older Adults
Johnson et al. (2021) “Good” [31]	United States (20-month period)	EXPT: Drivers (all ages) involved in MVC in Virginia Beach over a 20-month period COMP: Drivers (all ages) not involved in MVC that were sampled one week after crash cases in same location, road conditions and time of day	Blood and oral fluid; ELISA, LC-MS or GC-MS	On-scene data collection and police response	Crash rate of THC-positive drivers was significantly higher than that of sober drivers at ages 64 and 76 (F (1, 6757) = 3.8, *p* = 0.049; F (1, 6757) = 4.8, *p* = 0.028)
Kalantari Meibodi et al. (2015) “Poor” [29]	Iran (January–September 2012)	EXPT: Drivers (all ages) admitted to the emergency department following MVC COMP: Drivers (all ages) admitted to the emergency department for non-traumatic reasons	Self-report and urine (only drivers who claimed to have negative history of drug use); analytical technique not specified	Referral from emergency medical system following MVC	THC exposure was associated with lower odds of being involved in an MVC (OR, 95% CI 0.32 (0.11, 0.92)) in adults aged 55 and over
* Jørgenrud et al. (2018) “Poor” [32]	Norway (2016–2017)	EXPT: Drivers (all ages) with a positive history of MVC and speeding tickets in the last 12 months COMP: Drivers (all ages) with a negative history of MVC and speeding tickets in the last 12 months	Oral fluid; UPLC-MS/MS	Self-reported road traffic crashes	THC exposure was not significantly associated with odds of MVC involvement (OR, 95% CI 2.28 (0.12, 44.47)) in adults aged 50 and older
Mann et al. (2010) “Poor” [33]	Canada (2002–2005)	EXPT: Drivers (all ages) involved in MVCs that caused any kind of damage or injury in the past 12 months COMP: Drivers (all ages) not involved in MVCs in the past 12 months	Self-reported cannabis use	CAMH monitor self-report	Self-reported THC exposure in the last 12 months was not significantly associated with MVC risk among adults aged 55 and older (OR, 95% CI 1.18 (0.32, 4.36))

Abbreviations: EXPT = experimental group; COMP = comparison group; ELISA = enzyme-linked immunosorbent assay; LC-MS = liquid chromatography–mass spectrometry; GC-MS = gas chromatography–mass spectrometry; UPLC-MS/MS = ultraperformance liquid chromatography-tandem mass spectrometry. * Findings specific to older adults were not reported in the manuscript. This data was obtained from corresponding authors via data requests and analyzed separately to produce findings seen above.

**Table 3:** Characteristics and Results of the Included Culpability Studies.

Author (Year), Quality Rating	Country (Dates)	Group Characteristics	THC Exposure Ascertainment	Culpability Ascertainment	Key Findings in Older Adults
* Brubacher et al. (2019) “Good” [34]	Canada (2010–2016)	EXPT: Drivers (all ages) who were moderately injured in MVCs and deemed culpable for their crash COMP: Drivers (all ages) who were moderately injured in MVCs and deemed not culpable for their crash	Blood; broad-spectrum toxicological testing	Police crash reports	THC exposure did not have a significant effect on driver culpability (OR, 95% CI 1.21 (0.49, 2.96)) in adults aged 50 and over.
* Poulsen et al. (2014) “Good” [35]	New Zealand (2004–2009)	EXPT: Drivers (all ages) killed in MVCs who were deemed culpable for their crash COMP: Drivers (all ages) killed in MVCs who were deemed not culpable for their crash	Blood; immunoassay and LC-MS/MS	Police crash reports analyzed with Roberston and Drummer (1994) validated methodology	THC exposure was not significantly associated with culpability (OR, 95% CI 1.12 (0.12, 10.30)) in adults aged 50 and over.
* Chihuri & Li (2020) “Fair” [36]	United States (2011–2016)	EXPT: Drivers (all ages) killed in 2-vehicle MVCs who made ≥ 1 unsafe driver actions or errors COMP: Drivers (all ages) killed in 2-vehicle MVCs who made no unsafe driver actions or errors	Blood and urine; radioimmunoassay, LC-MS or GC-MS	Police reports and supporting documents	THC exposure did not significantly affect the risk of being deemed culpable for an MVC (OR, 95% CI 1.24 (0.94, 1.64)) in adults aged 50 and over.

Abbreviations: EXPT = experimental group; COMP = comparison group; UPLC-MS/MS = ultraperformance liquid chromatography-tandem mass spectrometry; LC-MS = liquid chromatography–mass spectrometry; GC-MS = gas chromatography–mass spectrometry. * Findings specific to older adults were not reported in the manuscript. This data was obtained from corresponding authors via data requests and analyzed separately to produce findings seen above.

## Data Availability

The data presented in this study are available in the article and Supplementary Materials.

## Supplementary Materials

The following supporting information can be downloaded at: https://www.mdpi.com/article/10.3390/brainsci13030421/s1, Figure S1: Funnel plot to explore publication bias for MVC risk; Figure S2: Funnel plot to explore publication bias for culpability.

## Appendix A

Search Strategies
Database: EBM Reviews-Cochrane Central Register of Controlled Trials <January 2021>
Search Strategy:
--------------------------------------------------------------------------------
1 exp Cannabis/(303)
2 cannabis.mp. (2655)
3 exp Cannabinoids/(826)
4 cannabinoid*.mp. (1133)
5 marijuana.mp. (1926)
6 exp Dronabinol/(737)
7 tetrahydrocannabinol*.mp. (1014)
8 nabilone.mp. (156)
9 exp Cannabidiol/(131)
10 cannabidiol*.mp. (700)
11 sativex.mp. (153)
12 or/1–11 (4763)
13 exp Automobile Driving/(821)
14 driv*.mp. (19485)
15 motorist*.mp. (15)
16 Safety/(3149)
17 Automobile Driver Examination/(23)
18 Licensure/(36)
19 ((driv* or motor* or safe*) adj3 (assess* or reassess* or screen* or licen* or relicen* or re-licen* or
test or re-test* or permit*)).mp. (55056)
20 Accidents, Traffic/(420)
21 crash*.mp. (688)
22 collision*.mp. (396)
23 accident*.mp. (21938)
24 or/13– 23 (95411)
25 12 and 24 (474)
Database: Embase Classic+Embase <1947 to 2021 Week 05>
Search Strategy:
--------------------------------------------------------------------------------
1 exp cannabis/(38661)
2 exp cannabis addiction/(10178)
3 exp cannabis derivative/(332)
4 cannabis.mp. (55670)
5 exp cannabinoid/(73223)
6 cannabinoid*.mp. (35085)
7 marijuana.mp. (19346)
8 exp tetrahydrocannabinol/(7048)
9 tetrahydrocannabinol*.mp. (14376)
10 exp nabilone/(1394)
11 nabilone.mp. (1451)
12 exp cannabidiol/(5459)
13 cannabidiol.mp. (6330)
14 exp nabiximols/(763)
15 sativex.mp. (727)
16 or/1–15 (94981)
17 exp car driving/(27133)
18 driv*.mp. (620343)
19 motorist*.mp. (729)
20 safety/(263615)
21 licensing/(24183)
22 driver licence/(2921)
23 ((driv* or motor* or safe*) adj3 (assess* or reassess* or screen* or licen* or relicen* or re-licen* or
test or re-test* or permit*)).tw. (93362)
24 exp traffic accident/(66650)
25 exp driver/(16113)
26 exp driving ability/(7496)
27 crash*.mp. (17733)
28 collision*.mp. (35924)
29 accident*.mp. (476435)
30 or/17– 29 (1429215)
31 16 and 30 (5535)
Database: Ovid MEDLINE(R) and Epub Ahead of Print, In-Process & Other Non-Indexed Citations, Daily
and Versions(R) <1946 to February 10, 2021>
Search Strategy:
--------------------------------------------------------------------------------
1 exp Cannabis/(9548)
2 cannabis.mp. (24434)
3 exp Cannabinoids/(14641)
4 cannabinoid*.mp. (24303)
5 marijuana.mp. (21437)
6 exp Dronabinol/(7197)
7 tetrahydrocannabinol*.mp. (7803)
8 nabilone.mp. (354)
9 exp Cannabidiol/(1823)
10 cannabidiol*.mp. (3715)
11 sativex.mp. (201)
12 or/1–11 (55974)
13 exp Automobile Driving/(20791)
14 driv*.mp. (491344)
15 motorist*.mp. (568)
16 Safety/(40479)
17 Automobile Driver Examination/(1424)
18 Licensure/(7134)
19 ((driv* or motor* or safe*) adj3 (assess* or reassess* or screen* or licen* or relicen* or re-licen* or
test or re-test* or permit*)).mp. (58034)
20 Accidents, Traffic/(44092)
21 crash*.mp. (14958)
22 collision*.mp. (39736)
23 accident*.mp. (200303)
24 or/13– 23 (798657)
25 12 and 24 (2680)
Database: APA PsycInfo <1806 to February Week 2 2021>
Search Strategy:
--------------------------------------------------------------------------------
1 exp Cannabis/(8919)
2 exp “Cannabis Use Disorder”/(492)
3 cannabis.mp. (12901)
4 exp Cannabinoids/(5766)
5 cannabinoid*.mp. (6588)
6 exp Marijuana/(3303)
7 exp Marijuana Usage/(3021)
8 marijuana.mp. (12183)
9 dronabinol*.mp. (1682)
10 exp Tetrahydrocannabinol/(1560)
11 tetrahydrocannabinol*.mp. (2594)
12 nabilone.mp. (90)
13 cannabidiol*.mp. (801)
14 sativex.mp. (71)
15 or/1–14 (26992)
16 exp Driving Behavior/(14895)
17 driv*.mp. (122720)
18 motorist*.mp. (498)
19 Safety/(14143)
20 exp Drivers/(6416)
21 ((driv* or motor* or safe*) adj3 (assess* or reassess* or screen* or licen* or relicen* or re-licen* or
test or re-test* or permit*)).mp. (15322)
22 exp Motor Traffic Accidents/(5889)
23 crash*.mp. (5702)
24 collision*.mp. (3585)
25 accident*.mp. (55110)
26 or/16 – 25 (193190)
27 15 and 26 (1347)
