# Supplementary material for: Risk of Motor Vehicle Collisions and Culpability among Older Drivers Using Cannabis: A Meta-Analysis
Source: Brain Sci. 2023 Feb 28;13(3):421. doi: 10.3390/brainsci13030421 (PMC10046364; doi:10.3390/brainsci13030421)
Supplement: Supplementary file 1 [file brainsci-13-00421-s001.zip › brainsci-2237574-supplementary.docx]

**Supplementary Figures**


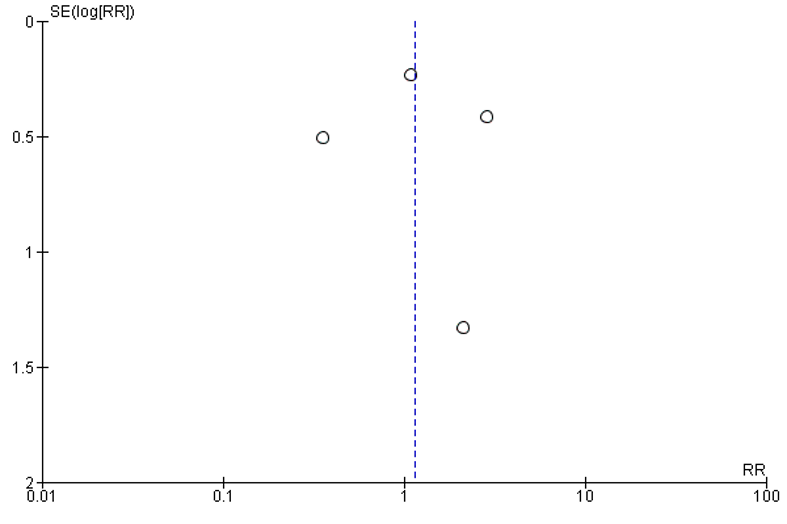


**Figure S1: Funnel plot to explore publication bias for MVC risk**


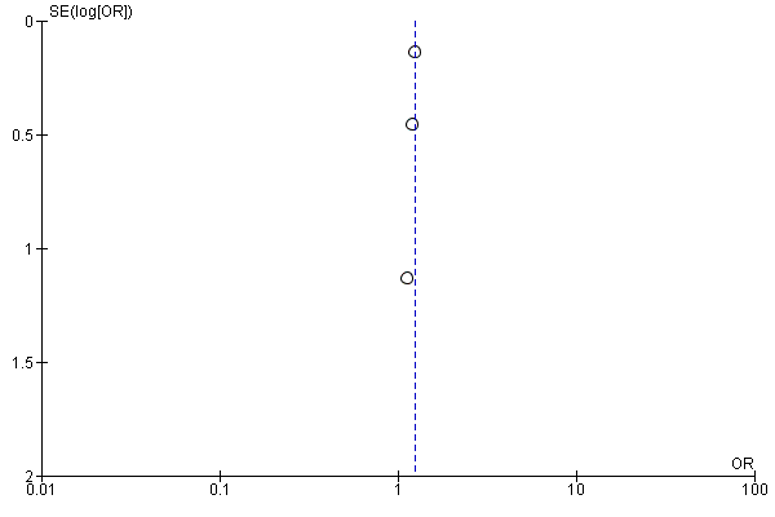


**Figure S2: Funnel plot to explore publication bias for culpability**
